# Retraction Note: Precision biochar yield forecasting employing random forest and XGBoost with Taylor diagram visualization

**DOI:** 10.1038/s41598-026-40191-5

**Published:** 2026-02-16

**Authors:** Sudhakar Uppalapati, Prabhu Paramasivam, Naveen Kilari, Jasgurpreet Singh Chohan, Praveen Kumar Kanti, Harinadh Vemanaboina, Leliso Hobicho Dabelo, Rupesh Gupta

**Affiliations:** 1 Department of Mechanical Engineering, Marri Laxman Reddy Institute of Technology and Management, Hyderabad, 500043 India; 2 Department of Research and Innovation, Saveetha School of Engineering, SIMATS, Chennai, Tamil Nadu 602105 India; 3 VEMU Institute of Technology, Chittoor, Andra Pradesh 517112 India; 4 School of Mechanical Engineering, Rayat Bahra University, Mohali, 140104 India; 5 University Center for Research and Development (UCRD), Chandigarh University, Mohali, 140413 Punjab India; 6 Department of Mechanical Engineering, Mattu University, P.O. Box 318, Mettu, Ethiopia; 7 Institute of Engineering and Technology, Chitkara University, Chitkara University, Rajpura, 140401 Punjab India; 8 Faculty of Engineering, Sohar University, 7119 Sohar, Oman

Retraction of: *Scientific Reports* 10.1038/s41598-025-91450-w, published online 27 February 2025

The Editors have retracted this Article.

Following publication, concerns were raised regarding the relevance of several cited references. Subsequent investigation revealed significant irregularities in authorship. The Authors were unable to provide satisfactory evidence to resolve these issues. As a result, the Editors no longer have confidence in integrity and the findings of the Article.

Prabhu Paramasivam and Praveen Kumar Kanti disagree with this retraction. Sudhakar Uppalapati, Naveen Kilari, Jasgurpreet Singh Chohan, Harinadh Vemanaboina, Leliso Hobicho Dabelo and Rupesh Gupta did not respond to correspondence regarding this retraction.

